# We need to talk about ‘bad’ resilience

**DOI:** 10.1136/bmjgh-2023-014041

**Published:** 2024-02-06

**Authors:** Dell D Saulnier, Stephanie M Topp

**Affiliations:** 1 Department of Clinical Sciences Malmö, Lund University, Lund, Sweden; 2 Department of Economics, University of Zambia, Lusaka, Zambia; 3 College of Public Health Medical and Veterinary Sciences, James Cook University, Townsville, QLD, Australia; 4 The University of Melbourne Nossal Institute for Global Health, Melbourne, Victoria, Australia

**Keywords:** Health systems, Health policy, Health systems evaluation

## Abstract

In this analysis, we argue against seeing health system resilience as an inherently positive concept. The rise in the popularity of health system resilience has led to its increasingly normative framing. We question this widely accepted perspective by examining the underlying assumptions associated with this normative framing of ‘good’ resilience. Our focus is on the risks of accepting the assumption, which can lead us to ignore the social nature of health systems and overlook the consequences of change if resilience is seen as a positive, achievable objective. Finally, we suggest that seeing resilience as a normative concept can be detrimental to health system policy and research, and encourage a critical rethinking of these assumptions so that we can maintain resilience’s usefulness for health systems.

**Summary box**

- There is a risk that health system resilience will no longer be useful if we continue seeing it as a normative concept.
- Resilience does not produce innately positive outcomes.
- Assuming that resilience is positive ignores the social nature of health systems.
- Framing resilience as an objective can also overlook the consequences of change.
- Resilience can still be useful if it used to explore health systems rather than evaluate them.

## Introduction

In their 2014 paper on resilience in development, Béné *et al* wrote, "It would be extremely useful to start talking about ‘bad' resilience’’.^1^ Resilience was consistently growing in popularity in development at the time, and the authors wrote that resilience ran the risk of becoming normative: a concept accepted as the correct development outcome, a vague metric against which to measure the success of development and an idealised objective for programmes to achieve.

Health system resilience is on a similar trajectory. The rise of health system resilience has been well documented before.^2 3^ Yet statements of the *need* for resilience, that systems *should* be resilient, or that health system resilience is *wanted*, are now common in research conclusions and in policy documents worldwide. Global institutions such as the WHO call for countries to ‘make health systems resilient for the achievement of UHC (universital health coverage) and health security’^4^ and the World Bank states that ‘the only way to prevent, prepare for, and manage [these] threats is by building resilient health systems’.^5^ These statements point at the very least to a discursive trend in which health system resilience is being used in normative fashion to frame assertions of what should be.

But what is health system resilience? While definitions vary, most agree that resilience is demonstrated in relation to something (i.e., shocks, everyday stresses) and indicated through maintaining some kind of core function.^6^ In keeping with this, we define health system resilience here in general terms: as the health system’s capacity to absorb, adapt or transform in order to maintain structures and functions when faced with shocks or stresses.^2^

Why then does it matter that global institutions and researchers are framing health systems resilience normatively? In short, a normative framing conflates a health system’s capacity with the outcomes or effects of that capacity.^7 8^ And while closely intertwined, these two things are not at all the same thing. The definition above reminds us that resilience is a capacity, whose purpose is to maintain health system function. However, nothing impels that capacity to operate in ways that produce innately positive outcomes. Normatively defined health system outcomes such as universal health coverage, equity of access and financial protection, are recognisably anchored to ethical principles such as fairness and justice. But the capacity to maintain function amidst shocks or stresses is just that: a capacity. It contains no guarantee of good (ethical) endpoints.^7^ As non-linear and self-organising, health systems can react and adapt in ways that produce both positive and negative effects. In other words, health system resilience is neither inherently good, nor inherently bad. It simply relates to the capacities that enable maintenance of certain functions and structure in response to a shock; the effects and outcomes of applying those capacities must be separately assessed.^9^

## ‘Bad’ and ‘good’ resilience

Despite these important distinctions, the concept of health system resilience is still frequently used to imply a normative outcome. Because of this, the potential for ‘bad’ resilience—by which we mean adaptation or transformation that maintains health system function but produces or perpetuates unfair, unjust or inequitable outcomes in the process—is seldom recognised.

A clear example is the ongoing health system challenge of achieving financial protection in healthcare. Despite a global commitment to universal health coverage, financial protection is still elusive, and indeed a rising trend of catastrophic health expenditures since 2012 indicates a growth rather than contraction of out-of-pocket payments to fund healthcare globally.^10^ These inequitable outcomes are frequently attributed to contextual limitations, such as restricted financial resources or inadequate commitment to financing reforms. Yet viewed from another perspective, the negative outcome of poor financial protection is itself the product of resilience in action. That is, the system’s (mal)adaptation to the shock of under-resourcing (and high demand) is precisely designed to introduce or increase out-of-pocket financing as a response that enables the system to maintain essential healthcare functions. The outcomes are highly inequitable, but system function is maintained.

Viewed like this, it is possible to see how health system resilience as a concept can explain (materially and ethically) negative outcomes, as well as positive ones. This is not to argue for low financial protection or against financing reforms. Rather, it is to highlight the importance of a clear-eyed framing of resilience, in which our assumptions about what it does are made explicit. If we are not clear on the different ways in which resilience capacity may manifest, we risk overlooking the all-important reasons for different types of response or resistance, missing the pathways to positive and negative outcomes of health systems strengthening initiatives that claim to target resilience.^8^

Indeed, equally useful questions to that of whether the health system is displaying resilience include: how and why do absorptive, adaptive and transformative capacities lead to changes in health system performance, outcomes and processes; and, to what ends are these capacities being employed by different individuals and groups within the health system?

The adaptive strategies used for the health workforce during COVID-19 are one example of the complexity in untangling assumptions about ‘good’ and ‘bad’ in resilience. For instance, in its report on building resilient health systems following COVID-19,^5^ the World Bank recommended that countries ‘develop, expand and sustain cadres of community health workers’ (CHWs) in order to improve outbreak detection and response at the community and primary healthcare level. Yet while CHWs were indeed critical to COVID-19 surveillance, control and education,^11^ research has shown that this impact was largely achieved at CHW’s own expense, in terms of money, time, resources and emotional well-being.^12^ In other words, the capacity to maintain community-level surveillance and education services in the face of the COVID-19 shock was based on maladaptive strategies that, intentionally or unintentionally, placed unfair expectations on this cadre of health workers. Although the World Bank report states that using CHWs to build resilient health systems would require professionalising the cadre and providing better supervision, skill building and compensation, it does not touch on the underlying mechanisms that would make developing, expanding and sustaining such a cadre possible, where previous initiatives to do the same have failed. Yet such mechanisms are essential if we want resilience capacities to evolve beyond the maladaptive reliance on vulnerable providers in order to maintain services during shocks. These mechanisms are the *how* that resilience can address.

## Who benefits from resilience?

A core argument against a normative framing of health system resilience is its continuing tendency to overlook to power. Overlooking power is to ignore a health system’s fundamental nature as a social, human system, dependent on the relationships between the people, groups, organisations and institutions that partially determine the system’s behaviour, and the values, judgements and norms that shape the health system’s context and the actors that make up the system.^13^ Critiques of health system resilience^6–8 14–16^ have pointed out that failing to understand aspects of power, such as agency, rational decision-making, and trust, erases what is done to enact (or not enact) change in order to maintain functions. Again, the emphasis remains on the outcome rather than the process and abilities that lead to it. This matters for normative applications of health system resilience because it can lead to assumptions that resilience confers equal impacts and benefits to all, and that actions ‘for resilience’ are somehow less influenced by power because they are done with the intent of strengthening resilience.

Health system changes may work for some yet have disproportionate or hidden impacts on others. Analysing health systems to draw conclusions on resilience or designing strategies and policies that are expected to create resilience means considering who may benefit and who may lose or suffer, either intentionally or otherwise.^9^ It means understanding for whom pressure may be relieved if a change occurs. For instance, Singapore’s health system response to COVID-19 was initially praised internationally for its resilience, attributed to its early success in adapting to maintain a variety of health services, through actions such as converting community buildings into community care facilities and keeping COVID-19 cases low with lockdowns and clear communication.^17^ However, migrant workers living in crowded dormitories who were outside the expected communication and healthcare channels and outside the community experienced disproportionately high infection rates early in the pandemic.^17^

Being aware of power also draws attention to the way certain adaptations may benefit some more powerful actors, despite negative effects or outcomes at the system level. For instance, global health initiatives, donors and international organisations—many based in the Global North and dominated by medical professionals—still wield outsized influence in health system agenda setting.^18 19^ Policy-makers in many countries health systems adapt to and prioritise externally set targets and agendas within their limited resources, in order to maintain donor relationships.^20^ While agreeing to conditional financing may maintain and even transform some health system functions, and may also provide short-term political benefit to its brokers, this is not the same as guaranteeing a stronger or fairer health system. Priorities set and investments made by more powerful actors can equally constrain as well as strengthen a health system’s ability to change during a crisis. For example, competing and conditional priorities imposed by international actors seriously fragmented West African nations’ response to the 2014–2016 Ebola outbreak and contributed to (rather than reduced) the scale of the crisis.^20 21^ Crucially, the power imbalance in this case was widely recognised as limiting the response. Although similar issues of power are being explored through arguments to decolonise global health,^22^ empirical work examining the links between adaptations made by powerful actors, and health and service outcomes via a resilience framing is still broadly lacking.^8^

Hand-in-hand with this perspective is the idea of agency—what do actors with different roles and at different levels have the power to actually do and how does this relate to the capacity for resilience? Research during floods in Cambodia showed that communities living in flood-prone areas were responsible for upholding the status quo, where communities were expected to continue obtaining facility-based maternal healthcare during floods and yet did not receive additional help or support to do so.^23^ With very little power to contradict expectations and forego facility visits, coping with healthcare needs during floods was pushed back onto the community. While this appeared to enhance the resilience of the health system during floods, it may have put it at risk. If the community was unable to cope during floods, the health system may not have the capacity to manage the new situation or consequences. In other words, the health system’s true capacity for resilience was both linked to and obfuscated by the community’s lack of power.

## The choice of outcomes and endpoints for resilience

Part of the problem in viewing all resilience capacity as producing positive outcomes arises from assuming that absorbing, adapting and transforming will have positive consequences or will at the very least minimise negative ones. But health systems are complex and adaptive, constantly changing to suit the environment around them, sometimes in unexpected ways.^24^ By focusing on only one or more expected, positive outcomes that are deemed to indicate ‘resilience’, we risk missing unexpected and unintended outcomes arising from the same changes.^2^

As an example, expanding telehealth services was touted as a positive adaptive strategy to COVID-19 lockdown shocks by removing physical risks and barriers associated with health services, thus helping to maintain services.^25 26^ It is obvious that scaling up telehealth services requires changing or redesigning service delivery processes, organisational practices and resource flows, all of which can lead to further change.^13^ However, there is a distinct mismatch between the assumed or implied effects of scaling up telehealth during a shock like COVID-19—better access to services, less risk for infection while seeking care, more efficient use of health system resources—and the consequences of making the adaptation itself. For instance, in its toolkit for a resilient health system, the WHO stated that telehealth services during crises can help address wider determinants of health and inequity and assure the quality of health services.^27^ But such assertions fail to question the ways in which contingent changes, such as new management practices or regulations, can influence outcomes such as inequity and quality of services. In lower-income countries, the new reliance on donor-supplied financing for telehealth services may disappear during coming funding cycles, threatening the stability of the services and thus their quality, reach and uptake.^28^ Reduced face-to-face contact as a result of new policies emphasising telehealth over in-person care may disadvantage rural, socially vulnerable or isolated groups, which in turn may have consequent impacts on their belief in the quality of care and trust in the health system, and thus equity.^29 30^ Thinking about consequences means focusing on why the adaptive change happens, what actual impacts (not a priori assumptions about impacts) it may have, and how these change the system as a whole.

## Is resilience useful?

Uncritically accepting resilience as a desirable outcome can blind us to its negative outcomes, yet acceptance and adoption of the ‘good resilience’ framing seem to prevail. Global initiatives and funding are more than ever focused on resilience as a measurable target for health systems strengthening. For example, in 2022, the World Bank released its Service Delivery Indicators health measurement tool that states it contains indicators that ‘are crucial to assess preparedness and resilience for epidemics, pandemics and shocks’,^31^ which includes input indicators such as guidelines, assessments and infrastructure. The Global Fund’s 2023–2028 strategy has made resilient health systems an objective in reaching the goal of ending AIDS, tuberculosis and malaria,^32^ and has dedicated US$9.2 billion to ‘building resilient health systems’ between 2024 and 2026.^33^ Most strikingly, a joint discussion paper on the Ukrainian health system’s recovery, authored by WHO, the United States Agency for International Development, the World Bank and the European Union (EU), have aligned reforms for health system resilience to epidemics and health emergencies with Ukraine’s possible accession into the EU.^34^ But, resilience is not the same thing as health system performance, as seen above^8^ and trying to measure and evaluate the resilience of the health system in this way contributes to a discursive tendency to use it in a normative manner.

Still, the concept of resilience does have an important place in health system research and practice if we stop seeing it as an outcome that needs to be achieved. The concept clearly offers a way of thinking about how to strengthen health systems based on the reality of the system’s behaviour. By using resilience to explore capacities and to ask why changes happen and how different capacities manifest, resilience can be used to explore and explain which processes, characteristics and relationships are supporting or hindering the system’s performance at a given point of change.^9^ This knowledge can be used to invest in underlying pathways more likely to lead to desirable health system behaviour; for example, pathways that support more equitable service access and reduced out-of-pocket expenditure. This could strengthen health systems while foregoing the assumption that ‘investing in resilience’ is equivalent to health system strengthening.

Resilience is a slippery term. As Béné *et al* and others have pointed out,^1 35^ resilience is useful in part because it is applied in multiple disciplines and fields of research. This means health system resilience can be used to help bridge discussion and bring together fields of work, from health system financing to planetary health to disease-specific programming, promoting a wider, renewed interest in thinking systemically about health systems and offering a deft conceptual tool in support of much needed explanatory research.^9^ Yet the intuitive and transdisciplinary applications of the term have also contributed to its increasingly normative framing in health research and can muddy the technical purpose it can serve for policy and practice as an explanatory and exploratory tool.^1 9^ If we can avoid assuming that resilience concepts that appear in other systems (eg, computing) can be uncritically (and we would argue, inappropriately) applied to social systems, then resilience should be able to add to what we know about how and why health systems function the way they do every day and during shocks.

## Conclusion

Normative views of resilience have already been critiqued in other fields such as development and disaster risk reduction.^36 37^ This paper is a call to reflect on the reality of ‘bad’ resilience and identify the pitfalls of the concept’s increasingly normative framing for health systems specifically. For resilience to retain its usefulness for global health systems research, policy and practice, we must understand what resilience can and cannot offer, refocusing our use of the concept away from a normative, evaluative approach and towards deeper exploratory work that helps us to understand how and why health systems function and perform as they do.

## Data Availability

No data are available.
